# Detection of slowly expanding lesions in people with multiple sclerosis using MRI: A scoping review

**DOI:** 10.1162/IMAG.a.1251

**Published:** 2026-06-11

**Authors:** Malo Gicquel, Anne Kerbrat, Benoit Combès

**Affiliations:** EMPENN Research Team, IRISA, CNRS-INSERM-INRIA, Rennes University, Rennes, France; Neurology Department, Rennes University Hospital, Rennes, France

**Keywords:** multiple sclerosis, chronic active lesions, slowly expanding lesions, chronic lesion expansion, chronic inflammation

## Abstract

Slowly expanding lesions (SELs) have recently been described in people with multiple sclerosis (pwMS). This pattern of slow lesion expansion, often too subtle to be identified visually, can be detected on conventional brain MRI scans and represents a candidate imaging biomarker of chronic inflammation. The present study undertakes a scoping review of articles reporting the detection of SELs in pwMS using MRI. Our main objectives were to summarize the different methods used to detect SELs, describe their frequency, examine their relationship with other markers of chronic inflammation, and assess their potential prognostic role. We also discuss the limitations of this biomarker and potential methodological improvements that should be explored before considering its translation into clinical routine. We identified 33 studies that detected SELs using longitudinal MRI acquisitions. Two distinct methods for detecting SELs were identified, each implemented with various parameter settings. SELs were detected in most pwMS (60–99%) across all disease phenotypes. SELs were more frequent than paramagnetic rim lesions (PRLs), and these two markers showed only modest co-localization. Several studies suggest that SELs may be a relevant marker for predicting disease progression and evaluating the effects of treatments on chronic inflammation. While SELs appear to be a promising biomarker in pwMS, current methods require further improvement and standardization, which limits inter-study comparability and hinders translation into clinical practice. Moreover, evidence regarding the sensitivity and specificity of SELs in identifying chronic active lesions in vivo remains limited.

## 1 Introduction

Multiple sclerosis (MS) is an inflammatory, demyelinating, and neurodegenerative disease of the central nervous system (Filippi et al., 2018) characterized by the presence of large demyelinated areas called MS lesions (Popescu & Lucchinetti, 2012). Along with this volumetric aspect, lesions should be characterized according to their current state of activity (active, inactive, or chronic active) (Kuhlmann et al., 2016). *Active lesions* are characterized by immune cells throughout the lesion area. *Chronic active lesions* (CALs) (Prineas et al., 2001) are former active lesions that feature a demyelinated inactive core with axonal loss and a demyelinating smoldering inflammation, with activated immune cells at their edge (Luchetti et al., 2018), while *inactive lesions* no longer feature these cells anywhere.

Being able to distinguish CALs from inactive and active lesions is of interest as histopathological analyses indicate that their prevalence is associated with disease severity (Popescu et al., 2017). Moreover, assessing the occurrence of CALs in patients could be valuable for therapeutic decision making, and the assessment of established and emerging treatments to reduce CAL burden.

However, while acute inflammation can be detected as new or substantially enlarging lesions between two T2-weighted/FLAIR MRI acquisitions, or using T1-weighted MRI acquired after the administration of a gadolinium-based contrast agent (Harris et al., 1991), detecting CALs in vivo remains difficult, especially in clinical settings. Currently, three main imaging biomarkers are used to detect CALs in vivo in pwMS (Bagnato et al., 2024). A first approach consists in using positron emission tomography (PET) targeting the 18-kDa translocator protein (TSPO), a protein overexpressed by activated microglia, which enables the detection of the active edge of CALs. TSPO PET helps advance the understanding of MS pathophysiology, but lacks accessibility due to its reliance on PET scans and radioactive tracers (Guido et al., 2025). The other two imaging biomarkers are based on MRI acquisitions, and are particularly promising for clinical practice (Scalfari et al., 2024). First, susceptibility MRI sequences can be used to detect accumulations of iron that may appear at the border of CALs, defining paramagnetic rim lesions (PRLs) (Toru Asahina et al., 2024). The second biomarker uses longitudinal T2-weighted/FLAIR and T1-weighted MRI acquisitions to detect the slow expansion that CALs may exhibit, such lesions are defined as “slowly expanding lesions” (SELs). PRLs have been thoroughly investigated, and their assessment is starting to be implemented in clinical settings. Reviews of SELs are still lacking, however, and the path toward the routine clinical use of SELs remains unexplored.

This review summarizes current knowledge on SELs, and presents the main difficulties to be overcome in order to translate them into clinical practice.

## 2 Method

### 2.1 Overall protocol

We conducted our review and reported our results in compliance with the Preferred Reporting Items for Systematic reviews and Meta-Analyses extension for scoping reviews (PRISMA-ScR) (Tricco et al., 2018).

### 2.2 Review objectives and inclusion criteria

For this scoping review, we intended to report results of studies detecting SELs using longitudinal MRI acquisitions. To find commonly used terms to describe expanding CALs, we used the Google scholar database to manually go through the list of all papers citing the first paper dedicated to SELs detection, Elliott et al. (2018). We then performed a systematic search of the PubMed electronic database using the following search term: *“multiple sclerosis” AND (SELs OR slowly expanding OR slowly evolving OR slowly enlarging OR chronic expansion) AND MRI*. This search was last performed on the 10th of September 2025. We exported the results as CSV and manually sorted the entries. We also conducted a search from the authors’ own collection of files citing Elliott et al. (2018).

We manually excluded sources that (1) did not detect expanding CALs in vivo, (2) were reviews, (3) were opinions, (4) were book chapters, (5) were thesis, (6) were not available for full text, (7) were preprints, (8) were not English.

## 3 Results

### 3.1 Query results

**Fig. 1.**
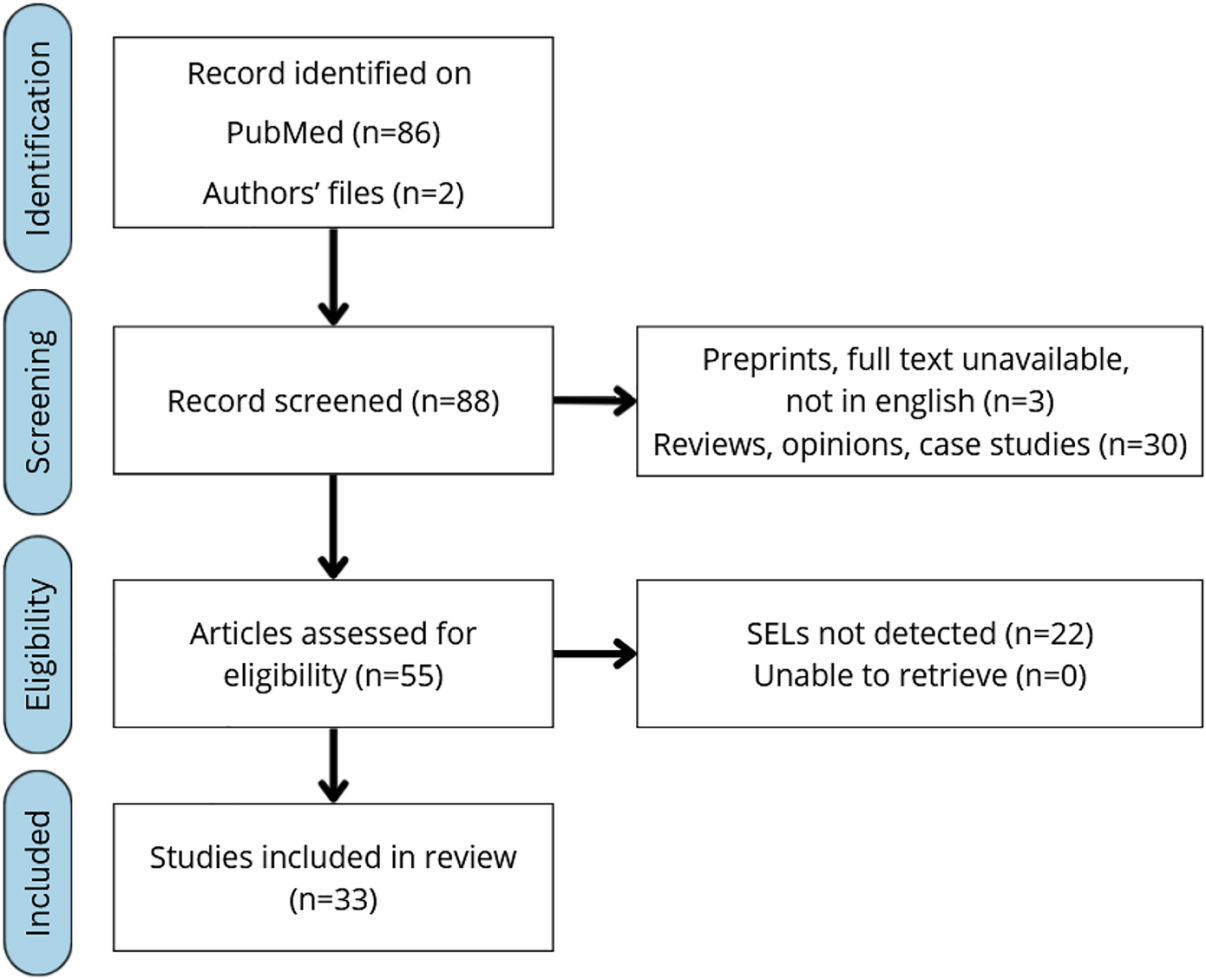
Flow diagram of review process

Our search resulted in 86 selected papers from PubMed to which were added 2 papers from the authors’ own files. Two papers that did not have full text available and one that was not in English were excluded directly on PubMed. Twenty papers were excluded because they were reviews, six because they were opinions, and four because they were case studies. After an investigation of the abstract and method sections, we excluded 22 eligible papers that did not detect SELs. One edge case was resolved by reviewer consensus. The process resulted in 33 selected papers. A PRISMA flow diagram is available in Figure 1.

### 3.2 Characteristics of the included studies

Overall, these papers encompass a wide diversity of populations and conditions. All studies were performed with European and American people with MS (pwMS), except one study that was performed with Asian pwMS (Yokote et al., 2024). Twenty-seven studies included pwRRMS, six pwPPMS, and eight pwSPMS. All studies excluded patients under 18 years old, except one study (Fadda et al., 2024). The number of patients included ranged from 15 to 2388.

All studies were conducted with 3T or 1.5T MRI systems, except one study that used a 7T MRI system (Huerta et al., 2024). All studies included 2D or 3D FLAIR/T2w acquisitions. The vast majority of studies used MPRAGE T1w acquisitions, although two studies (Huerta et al., 2024; Ravano et al., 2024) used MP2RAGE T1 instead.

### 3.3 Methods to characterize slowly expanding lesions

SEL detection requires at least two acquisitions from a given patient performed at different times. The shortest period used between the baseline and the last follow-up was 48 weeks (Arnold et al., 2024) and the largest was 7 years (Klistorner et al., 2025). Two alternative approaches to detect SELs have been implemented and are described in this section.

#### 3.3.1 Method based on segmentation analysis

**Fig. 2.**
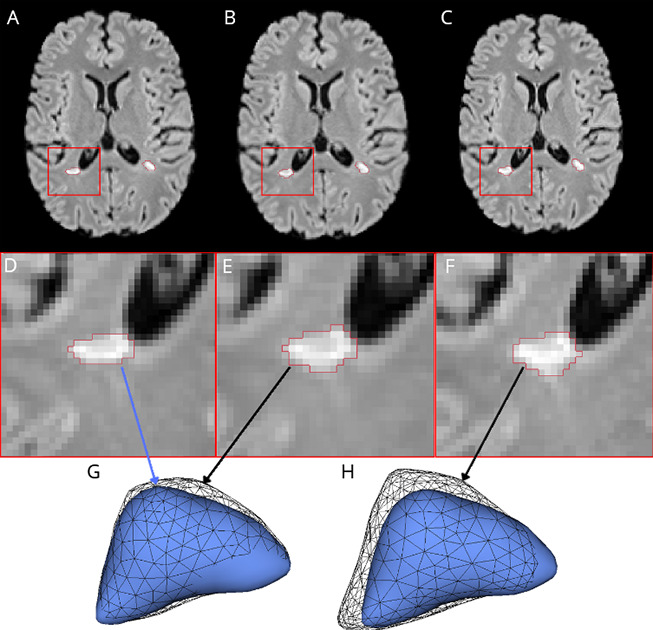
MRI-defined slowly expanding lesions (SELs) based on segmentation analysis. FLAIR MRI axial slice and T2 lesions contoured in red at (A) baseline, (B) 12-month follow-up, and (C) 24-month follow-up, all rigidly registered to an undisplayed reference scan. A region of interest corresponding to the red box, with an MS lesion at its center at (D) baseline, (E) 12-month follow-up, and (F) 24-month follow-up. A 3D representation of the evolution of the MS of the region of interest, with the blue surface being the lesion at baseline (volume 211 mm^3^) and the black wire frame being the lesion at (G) 12-month follow-up (volume 254 mm^3^) and (H) 24-month follow-up (volume 290 mm^3^).

This method was used in 11 of the 33 (33%) reviewed papers (Klistorner, Barnett, Graham, et al., 2022; Klistorner, Barnett, & Klistorner, 2022; Klistorner, Barnett, Parratt, Yiannikas, & Klistorner, 2024a, 2024b; Klistorner, Barnett, Wang, et al., 2024; Klistorner, Barnett, Yiannikas, et al., 2022; Klistorner, Barnett, et al., 2022; Klistorner et al., 2021, 2025; Moog et al., 2022; Ravano et al., 2024), among which 9 were from the same research group. It requires at least two acquisitions, usually performed at least 1 year apart, on which white matter lesions can be segmented on T2w or FLAIR sequences. Figure 2 summarizes this approach, which can be described with the following four successive steps:

1. **Delineating the lesions:** Estimate the T2 white matter lesions segmentation mask at each time point, using an existing deep-learning model in a fully automated way (e.g. Ravano et al., 2024) or in a semi-automated way adding an expert’s correction (e.g. Klistorner et al., 2021).
2. **Post-processing of lesion masks:** New confluent lesions may be excluded from the analysis (Klistorner et al., 2021). Gadolinium-enhancing lesions and new lesions can also be removed to reduce the influence of acute inflammatory activity (Klistorner, Barnett, Graham, et al., 2022; Klistorner, Barnett, & Klistorner, 2022; Klistorner, Barnett, Parratt, Yiannikas, & Klistorner, 2024a, 2024b; Klistorner, Barnett, Wang, et al., 2024; Klistorner, Barnett, Yiannikas, et al., 2022; Klistorner, Barnett, et al., 2022; Klistorner et al., 2021).
3. **Inter-session lesion mapping:** Map each lesion in the baseline mask to its follow-up counterpart, typically by registering the two MRIs.
4. **Longitudinal volumetric analyses:**
  4.1. **Lesion classification:** Categorize each lesion into “stable”, “expanding”, and potentially “shrinking”, depending on volume change over time.
  4.2. **Continuous chronic tissue expansion analysis:** After the exclusion of shrinking lesions, global chronic tissue expansion analysis can be performed without further classification by measuring the total longitudinal volume change of all remaining lesions. This provides a patient-level continuous metric of chronic lesion expansion.

The lesion classification step is critical and can be performed in two main ways. When more than two time steps are available, different strategies to integrate the information from the different intervals can be used. One way involves considering each successive time interval independently and classifying each lesion for a given time interval depending of its annual rate of volume change. In all studies (Klistorner, Barnett, Graham, et al., 2022; Klistorner, Barnett, & Klistorner, 2022; Klistorner, Barnett, Parratt, Yiannikas, & Klistorner, 2024b; Klistorner, Barnett, Wang, et al., 2024; Klistorner, Barnett, Yiannikas, et al., 2022; Klistorner, Barnett, et al., 2022; Klistorner et al., 2021) except one study (Klistorner, Barnett, Parratt, Yiannikas, & Klistorner, 2024a), a minimal annual volume change of 2.5% is used to differentiate expanding from stable lesions, this threshold was chosen by investigating the variability of lesional volumes on test–retest manual segmentations (Klistorner et al., 2021). The exception used 4%, to match one of the thresholds used by Elliott et al. (2018). A second way (Ravano et al., 2024) consists in considering all time points at once by detecting baseline lesions whose successive volumes experienced a sufficiently positive linear trend. This was assessed using a numerical simulation approach in which random fluctuations were calibrated by deducing the variability in segmentation volumes measurements from segment–resegment strategies and scan–rescan patient acquisitions.

When using segmentation analysis, the detected lesions are often referred to as “expanding lesions” or “chronic lesions” instead of “SELs”.

#### 3.3.2 Method based on deformation analysis

**Fig. 3.**
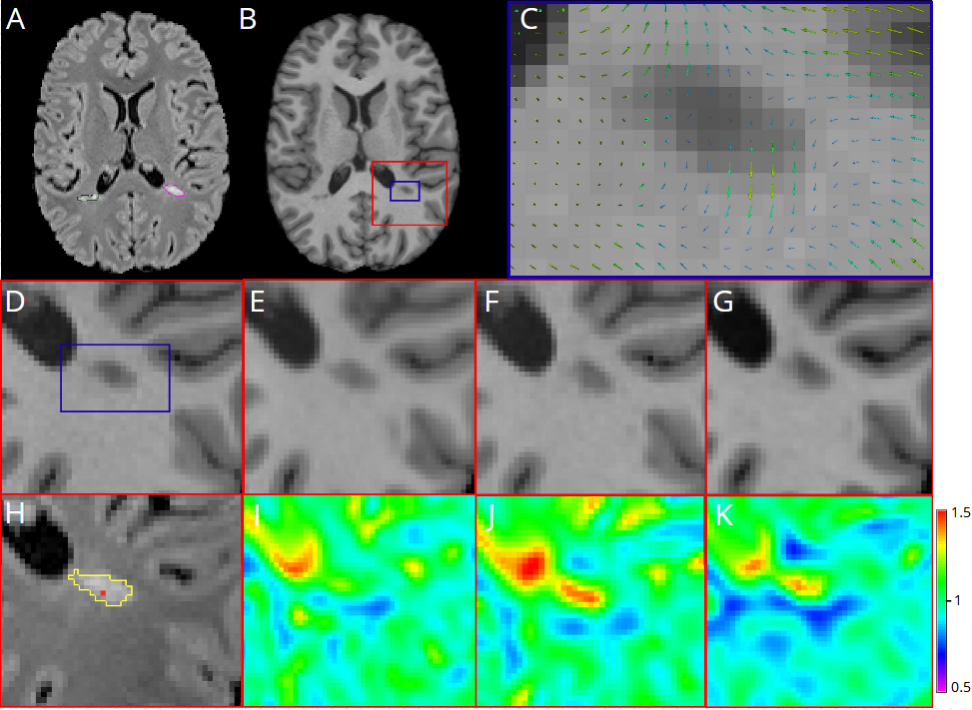
MRI-defined slowly expanding lesions (SELs) based on longitudinal deformation analysis. (A) FLAIR MRI axial slice and T2 lesions contoured at baseline, (B) T1-weighted MRI axial slice at baseline, and (C) deformation vector field from baseline to 36 months and baseline T1-weighted MRI axial slice in a region of interest corresponding to the blue box. A region of interest corresponding to the red box, with an MS lesion at its center, (D) T1-weighted MRI axial slice at baseline, (E) 12 months, (F) 24 months, and (G) 36 months. (H) FLAIR acquisition at baseline with an SEL contour in yellow and its core in red. Jacobian determinant maps in the region of interest representing relative volume growth at (I) 12 months, (J) 24 months, and (K) 36 months. Pixels in green to red (J>1 ) indicate a local expansion; pixels in blue indicate a local contraction.

This method was used in 22 of the 33 (67%) reviewed papers (Arnold et al., 2024; Bar-Or et al., 2023; Beynon et al., 2022; Calvi, Carrasco, et al., 2022; Calvi, Tur, et al., 2022; Calvi et al., 2023, 2024, 2025; Cross et al., 2024; De Meo et al., 2025; Elliott et al., 2018, 2019, 2020, 2023; Fadda et al., 2024; Huerta et al., 2024; Jiang et al., 2023; Nakamura et al., 2024; Preziosa et al., 2021, 2022; Vavasour et al., 2025; Yokote et al., 2024) by various teams. This method relies on detecting regions of existing T2 white matter lesions (T2WML) that show a consistent and concentric local time expansion. It was developed by Elliott et al. (2018). Figure 3 shows the main steps of this method. In principle, it requires brain 2D or 3D T1w/MP2RAGE and T2w/FLAIR acquisitions at three time points or more and a baseline T2 white matter lesions segmentation mask. The method can be summarized as follows:

1. **Pre-processing:** Estimate the T2 white matter lesions segmentation mask at baseline, using an existing deep-learning model in a fully automated way (e.g. Yokote et al., 2024) or in a semi-automated way adding an expert’s correction (e.g. Calvi, Tur, et al., 2022; Elliott et al., 2018; Preziosa et al., 2021). Resample to an isotropic space. Cross-sectional and longitudinal linear registrations to the baseline FLAIR scan for global alignment.
2. **Post-processing of lesion masks:** Potentially remove gadolinium-enhancing lesions and new lesions to reduce the influence of acute inflammatory activity.
3. **Estimating the deformation fields:** Perform non-linear registrations between the baseline and each of the follow-up scan to calculate one deformation field per follow-up, using both the T1w and the T2w/FLAIR images.
4. **Estimating local volume change:** Estimate the determinant of the Jacobian of the computed fields and resample it to the initial T2w/FLAIR scan spatial resolution.
5. **SELs detection:** Estimate SEL regions from the baseline T2WML map and the Jacobian determinant of the last follow-up.
6. **Post-processing of SELs:** Remove SELs smaller than 10 voxels in size for more reliability.

In this process, two main choices are critical. First, Step 4 necessitates the choice of a non-linear registration algorithm. Most studies (20 of 22) used “SyN” non-linear registration from ANTs (Avants et al., 2008), using both the T1w and the T2w/FLAIR images at the same time (Arnold et al., 2024; Bar-Or et al., 2023; Beynon et al., 2022; Calvi, Carrasco, et al., 2022; Calvi, Tur, et al., 2022; Calvi et al., 2023, 2024, 2025; Cross et al., 2024; Elliott et al., 2018, 2019, 2020, 2023; Fadda et al., 2024; Jiang et al., 2023; Nakamura et al., 2024; Vavasour et al., 2025; Yokote et al., 2024) or the MP2RAGE images only (Huerta et al., 2024). The remaining studies (2 of 23) used serial longitudinal registration from SPM12 (Ashburner, 2013), using merged T1w and T2w images (Preziosa et al., 2021, 2022). Second, Step 5 is also critical and can be performed in three ways. The first one, used by 14 studies, first identifies the SEL cores/clusters as voxels of the T2WML segmentation mask, where the local expansion between the baseline and the last follow-up is greater than a threshold of volume increase per year *JE*_1_. Then, starting from these SEL clusters, SELs are iteratively extended to all neighboring voxels where the local expansion is larger than a second smaller yearly threshold *JE*_2_ to determine the final limits of the SELs. This two stages process is used to ensure that distinct expansions are considered as distinct lesions (Elliott et al., 2018). This approach however loses the biological interpretation provided by T2 lesion delineation. All but one study (Yokote et al., 2024) used a cluster threshold *JE*_1_ of 12.5% per year and a second threshold *JE*_2_ of 4% per year (Arnold et al., 2024; Bar-Or et al., 2023; Beynon et al., 2022; Cross et al., 2024; Elliott et al., 2018, 2019, 2020, 2023; Fadda et al., 2024; Huerta et al., 2024; Jiang et al., 2023; Nakamura et al., 2024; Vavasour et al., 2025), initially set empirically through visual assessments by Elliott et al. (2018). The study that used a different threshold (Yokote et al., 2024) empirically set *JE*_1_ = 6% as they studied a population with a mild MS activity, on which no SELs would have been detected otherwise. The second way consists in identifying SELs among the T2 lesions that contain voxels classified as SELs in the previous procedure (Preziosa et al., 2021, 2022) and defining the number of SELs as the number of SELs clusters, to separate distinct expansions within T2 lesions. One study (Preziosa et al., 2021) investigated the effect of the clustering threshold *JE*_1_ on the mean number of SELs and observed that the choice of *JE*_1_ does not affect the prevalence ratio between their groups of patients. The last way SELs can be detected involves selecting individual T2 lesions in which the mean Jacobian determinant is higher than a fixed threshold. Two teams set this threshold to 0% (Calvi, Carrasco, et al., 2022; Calvi, Tur, et al., 2022; Calvi et al., 2023, 2024, 2025; De Meo et al., 2025), effectively considering the expansion estimator as error free.

Once individual SELs have been identified, each can be attributed a confidence score, reflecting the expected constancy and concentricity of their expansion over time (Elliott et al., 2018). These scores, respectively, assess their constant gradual growth over time using the Jacobian determinant obtained for each follow-up in Step 3 and the concentric pattern of outward radial expansion in the Jacobian determinant associated with the last follow-up. These scores are combined into a single one through a normalization process relying on cohort-level statistics. Information on the calculation of the score is given in Appendix A.1. SELs with a sufficient score are “definite SELs”; the others are “possible SELs”. Results of comparisons between definite and possible SELs are available in Appendix A.2. “Non-SELs” T2 lesional regions that devoid of any SELs detection (Elliott et al., 2020; Preziosa et al., 2021).

#### 3.3.3 SEL metrics

Once SEL regions have been defined by any of these methods, metrics can be extracted.

**SELs volume and count:** In most studies, the metrics typically consist of the number of SEL clusters and/or their volumes. These quantities can then be kept as absolutes or normalized to the total patient lesions count/volume.

**SELs volume change:** The expansion speed of SELs (expressed in $mm^{3}\text{​}/\text{​}year$ ). For the method based on deformation analysis, it can be characterized using the local expansion, as measured by the Jacobian determinant within each SEL (Elliott et al., 2018, Supplementary).

**SEL and non-SEL region of interest:** Many studies also use SEL and non-SELs lesion masks as regions of interest to extract tissue-integrity measurements, typically measured using quantitative MRI sequences. Measures of tissue integrity can be quantified at baseline and by their change between two time points. Interestingly, the segmentation-based method provides a delineation of the regions of interest at both baseline and follow-up.

**Combined metrics:** Combined metrics also exist, such as the progressive volume/severity index (PVSI) (Klistorner, Barnett, & Klistorner, 2022; Klistorner, Barnett, Parratt, Yiannikas, & Klistorner, 2024b; Klistorner, Barnett, Wang, et al., 2024), which is the volume of SELs multiplied by a measure of tissue integrity change over time.

In practice, chronic active lesions do not exhibit any minimal or expected expansion rate. Furthermore, expansion measurements are prone to errors. By contrast to directly studying continuous measurements, reducing lesion expansion dynamics to categories through the use of arbitrary thresholds, therefore, leads to less informative SELs measures that may limit their usefulness for further analysis.

**Statement:** SELs are a candidate biomarker of chronic inflammation, computed from conventional MRI sequences, and acquired longitudinally over at least two time points. They can be defined as enlarging white matter T2 lesions in segmentation maps or as areas of pre-existing T2 lesions that show local expansion in T2- and T1-weighted MRIs. To date, no study has directly compared these two approaches, and the impact of using one method over the other remains unclear. Segmentation-based methods have primarily been applied to patients followed over multiple years, whereas deformation-based methods have been used over shorter time intervals, as they may capture subtler changes at a sub-voxel scale. It should also be noted that inter-site variability analyses of brain atrophy measurements have shown that segmentation-based methods are less reliable than registration-based approaches for quantifying longitudinal brain volume loss in pwMS (Durand-Dubief et al., 2012). In deformation-based methods, the SEL scoring strategy may require further development, particularly regarding its dependence on cohort-specific distributions. These observations further underscore the need for studies directly comparing the results of these different methods.

More generally, this biomarker has various methodological limitations. First, there is no standardized method available. Consequently, critical parameters may vary from one study to another, resulting in low inter-study comparability. The most critical parameters of the segmentation-based method are the segmentation algorithm and the classification method to distinguish enlarging lesions from stable/shrinking lesions, especially the volume-expansion threshold. The most critical parameters of the deformation-based method are probably the two volume-expansion thresholds $JE_{1}$ and $JE_{2}$, and the non-linear deformation algorithm and its associated hyperparameters. In the absence of ground truth detection of CALs, assessing the specificity and sensitivity of SELs to CALs is difficult, making the validation of the method and the parameters harder. Second, since brain volumes are not necessarily preserved between different scanners, scanner changes may significantly influence SEL estimation by artificially increasing or decreasing the estimated volume expansion. The impact of this issue has not been investigated for either method yet, but it could be an important issue in clinical settings because of the frequent scanner changes.

### 3.4 Demographics of SELs

#### 3.4.1 SELs frequency according to disease phenotype

Patients showing at least one SEL are referred to as SEL+ patients and the others as SEL- patients. Studies found 60% to 99% of SEL+ pwMS (Beynon et al., 2022; Calvi, Carrasco, et al., 2022; Calvi et al., 2023, 2025; Elliott et al., 2018, 2019, 2020; Klistorner et al., 2021; Moog et al., 2022; Preziosa et al., 2021). One study on Japanese pwRRMS (with a milder disease activity) found only 35% of SEL+ patients (Yokote et al., 2024). With regard to disease phenotypes, studies found 60% to 94% of SEL+ pwRRMS (Elliott et al., 2018, 2020; Klistorner et al., 2021; Moog et al., 2022; Preziosa et al., 2021), 79% to 99% of SEL+ pwSPMS (Beynon et al., 2022; Calvi, Carrasco, et al., 2022; Elliott et al., 2020), and 68% to 85% of SEL+ pwPPMS (Beynon et al., 2022; Elliott et al., 2019). Moreover, SELs represented 21% to 46% of T2 lesions in pwMS (Calvi, Carrasco, et al., 2022; Calvi et al., 2023, 2025; Huerta et al., 2024; Klistorner et al., 2021). Interestingly, one study observed a continuum of change in the patient-wise SELs expansion distribution, making the dichotomization between expanding and stable patients difficult (Klistorner et al., 2025).

A few studies compared SELs in various disease phenotypes: RRMS versus PPMS (Elliott et al., 2018) and RRMS versus SPMS (Elliott et al., 2020). Compared with pwRRMS, pwPPMS had a higher SELs count, relative and absolute SELs volume, but the same proportion of patients had at least one SEL in both groups (72% versus 68%) (Elliott et al., 2018). Similarly, compared with pwRRMS, pwPPMS had a higher median SELs count and volume but a similar percentage of patients had at least one SEL in both groups (89% versus 83%). However, the number and proportion of SELs were no longer significantly different between groups when accounting for baseline T2 lesion volume, age, and sex (Elliott et al., 2020).

#### 3.4.2 Other factors associated with the occurrence of SELs

SELs were reported to be more frequent in male patients (Calvi, Tur, et al., 2022; Klistorner et al., 2025), and to be positively associated with patient age (Calvi, Tur, et al., 2022; Klistorner et al., 2025) and disease duration (Calvi, Tur, et al., 2022). These factors are also predictors of disease progression and are known to be associated with CALs prevalence in histopathological studies (Frischer et al., 2015).

#### 3.4.3 SELs location

Various studies used a brain atlas to generate a spatial distribution map of SELs (Calvi, Carrasco, et al., 2022; Elliott et al., 2018; Nakamura et al., 2024; Vavasour et al., 2025). These studies consistently demonstrated that SELs are preferentially located in periventricular areas, similar to T1/T2 lesions in general. A closer inspection (Klistorner, Barnett, Graham, et al., 2022) showed that lesion expansion was inversely proportional to the distance to the ventricles.

Two studies investigated the directions along which SELs expand (De Meo et al., 2025; Moog et al., 2022). Enlarging T2 lesion centroids were transitioning over time to the cortex, while contracting T2 lesion centroids were transitioning toward the center of the scan. This transition was not due to shape changes and was not observed in expanding small vessel disease lesions (Moog et al., 2022). One group used a DTI template to estimate white matter tract directions and a SWI template to estimate a vein atlas and observed that WML expansion occurred preferentially along white matter tracts and veins (De Meo et al., 2025).

#### 3.4.4 SELs in other neurological pathologies

Only a few studies applied SEL-detection methods to other neurological disorders to study the specificity of SELs to MS. First, when comparing pediatric-onset multiple sclerosis (POMS) and MOG-associated disease (MOGAD), SELs were almost absent in MOGAD patients, as only 1 SEL was found in 14 patients, while 70 SELs were found in 19 pwPOMS (only 3 did not have SELs) (Fadda et al., 2024).

A second study compared pwRRMS with patients with small vessel disease (SVD). The authors detected expanding lesions in 28 of 35 pwRRMS, while all 12 pwSVD had expanding T2 lesions (Moog et al., 2022). No detail regarding their frequency within patients was reported. However, the pattern in lesion enlargement was different: SELs had a greater displacement over time than enlarging SVD lesions, and unlike SVD lesions, expanding MS lesion centroids were moving over time toward the cortex (Moog et al., 2022).

**Statement:** SELs are a common feature of MS, found in most patients and in every disease phenotype, although they represent more lesions and lesional volume in pwPMS. They are preferentially located near the ventricles. Inter-study comparison of SELs prevalence is made difficult by the variations in method and expansion thresholds used. SELs are more common in males, older patients, and patients with a longer disease duration. Data regarding potential comorbidities associated with SELs, such as the presence of cardiovascular risk factors, are currently not available. The detection of SELs does not appear to be entirely specific to MS. This point remains to be confirmed by further studies.

### 3.5 Associations between SELs and other markers of chronic inflammation

CALs can be detected using various techniques, the most commonly used consists in detecting the iron-laden microglia ring that may be present at their border. The paramagnetic properties of iron allow the detection of “paramagnetic rim lesions” using susceptibility MRI. PRLs seem to be very specific, but moderately sensitive to CALs (Hemond et al., 2025). PRLs have previously been shown to enlarge often and significantly more than non-PRLs (Absinta et al., 2016; Dal-Bianco et al., 2016).

The association between SELs and PRLs has been assessed, and it was found that SELs and PRLs have a moderate co-localization (Calvi et al., 2023; Elliott et al., 2023). In one study, 616 SELs and 80 PRLs (for a total of 1,492 lesions) were found in 61 pwRRMS. Among them, 92% had at least one SEL and 56% had at least one PRL; 7% of SELs were PRLs, versus 4% for non-SELs (Calvi et al., 2023). In another study, 267 SELs and 119 PRLs were found in 41 pwMS; 70% of patients had at least two SELs, 59% had at least one PRL, 39.5% of PRLs were SELs, while 17.2% of SELs were PRLs (Elliott et al., 2023).

Characterizing lesions using both markers can help further identify the lesion type in terms of the accumulation of tissue damage over time, as PRL+/SEL- had more baseline tissue damage than SEL+/PRL-, but this damage was stable over time, whereas SEL+/PRL- showed ongoing tissue destruction and SEL+/PRL+ accumulated more tissue damage than SEL+/PRL- (Elliott et al., 2023). Moreover, the group of patients with at least one SEL and one PRL had the highest lesion count and volume, and EDSS progression (Calvi et al., 2023).

**Statement:** Studies investigating the association between SELs and other markers of chronic inflammation are scarce, and have reported only the association between SELs and PRLs. SELs are more frequent than PRLs. They show a minimal co-localization, indicating that they are likely to reflect distinct pathological processes and they could give complementary information about CALs. This could be due not only to different lesion phenotypes or different time frames within the life cycle of chronic lesions, but also to the impact of more technical factors. Thus, lesion expansion could be caused by other factors such as neurodegeneration and blood–brain barrier leakages. The associations between SELs and TSPO PET and between SELs (assessed on antemortem longitudinal MRI) and anapathology, two other markers of chronic inflammation, are still unknown.

### 3.6 Associations between SELs and MRI measures of microstructural damage

To assess tissue damage in SELs, various quantitative or semi-quantitative MRI sequences and derived metrics have been used. In the next paragraphs, the results obtained using these metrics will be grouped according to their predominant association with axonal damage or demyelination, although a given metric is not fully specific to a single damage component.

#### 3.6.1 Microstructural damage in SELs

Results assessing the accumulation of microstructural damage in SELs are detailed in Table 1.

**Table 1.** White matter tissue damage characterization associated with chronic inflammation.

Metric used	Comparison	Population of interest	Effect
MTR (demyelination)	SELs to non-SELs	RR (N=52 )	Worse values at baseline (Preziosa et al., 2021)
		RR (N=242 ), SP (N=57 )	Worse values at baseline (Elliott et al., 2020)
		RR (N=50 )	Worse values at baseline (Vavasour et al., 2025)
		PP (N=107 ), SP (N=88 )	Worse values at baseline (Nakamura et al., 2024)
	SELs over time	RR (N=52 )	Stable over 2 years (Preziosa et al., 2021)
		RR (N=242 ), SP (N=57 )	Worsening over 72 weeks (Elliott et al., 2020)
		RR (N=83 )	Worsening over 3.2 years (Calvi, Tur, et al., 2022)
		RR (N=50 )	Worsening over 96 weeks and 192 weeks (Vavasour et al., 2025)
		PP (N=107 ), SP (N=88 )	Worsening over 96 weeks (Nakamura et al., 2024)
	Definite SELs to non-SELs	SP (N=106 )	Worse values at baseline (Calvi, Carrasco, et al., 2022)
	Definite SELs over time	SP (N=106 )	Worsening over 96 weeks (Calvi, Carrasco, et al., 2022)
	Definite SELs to possible SELs	RR (N=83 )	Worse values in definite SELs and more variability in possible SELs (Calvi, Tur, et al., 2022)
	SELs, SPMS vs RRMS	RR (N=242 ), SP (N=57 )	Worse values at baseline in SPMS and same change over 72 weeks (Elliott et al., 2020)
	NAWM in SEL+ patients to NAWM in SEL- patients	RR (N=50 )	More voxels had worse values and more deterioration over 96, 192 weeks (Vavasour et al., 2025)
DTI-RD (demyelination)	SELs to non-SELs	RR (N=242 ), SP (N=57 )	Worse values at baseline (Elliott et al., 2020).
		RR (N=50 )	Worse values at baseline (Klistorner, Barnett, Yiannikas, et al., 2022)
	SELs over time	RR (N=242 ), SP (N=57 )	Worsening over 72 weeks (Elliott et al., 2020)
		RR (N=50 )	Worsening over 5 years (Klistorner, Barnett, Yiannikas, et al., 2022)
	SELs periplaque to non-SELs periplaque	RR (N=50 )	Worse values at baseline (Klistorner, Barnett, Yiannikas, et al., 2022).
	SELs, SPMS vs RRMS	RR (N=242 ), SP (N=57 )	Worse values at baseline in SPMS and same change over 72 weeks (Elliott et al., 2020)
Apparent water myelin fraction (demyelination)	SELs to non-SELs	RR (N=15 )	Worse values at baseline (Huerta et al., 2024)
		RR (N=50 )	Worse values at baseline (Vavasour et al., 2025)
	SELs over time	RR (N=15 )	Worsening over 1 year (Huerta et al., 2024)
		RR (N=50 )	Worsening over 96 weeks and 192 weeks (Vavasour et al., 2025)
	Non-SELs in SEL+ patients to non-SELs in SEL- patients	RR (N=50 )	Worse baseline values (Vavasour et al., 2025)
	NAWM in SEL+ patients to NAWM in SEL- patients	RR (N=50 )	More voxels had worse values and more deterioration over 96, 192 weeks (Vavasour et al., 2025)
Bound pool fraction (demyelination)	SELs to non-SELs	RR (N=15 )	Worse values at baseline (Huerta et al., 2024)
	SELs over time	RR (N=15 )	Worsening over 1 year (Huerta et al., 2024)
T2 relaxometry (demyelination)	SELs periplaque to stable lesions periplaque	RR (N=221 ), SP (N=32 )	Worse baseline values and more variability (Ravano et al., 2024)
DTI-AD (axonal damage)	SELs to non-SELs	RR (N=50 )	Worse values at baseline (Klistorner, Barnett, Yiannikas, et al., 2022)
	SELs over time	RR (N=50 )	Worsening over 5 years (Klistorner, Barnett, Yiannikas, et al., 2022)
	Periplaque SELs to periplaque non-SELs	RR (N=50 )	Similar values at baseline (Klistorner, Barnett, Yiannikas, et al., 2022)
Fractional anisotropy (axonal damage and demyelination)	Definite SELs to possible SELs and non-SELs	RR+PP+SP (N=130 )	Worse baseline values (Calvi et al., 2025)
	Definite SELs core and perilesional areas over time	RR+PP+SP (N=130 )	Consistent decline over 2 years, especially in the core, non-SELs showed the opposite pattern (Calvi et al., 2025)
Neurite density (axonal damage)	SELs to non-SELs	RR (N=15 )	No difference at baseline (Huerta et al., 2024)
	SELs over time	RR (N=15 )	No worsening over 1 year (Huerta et al., 2024)
T1w (axonal damage)	SELs to non-SELs	RR (N=52 )	Worse values at baseline (Preziosa et al., 2021)
		RR (N=1334 ), PP (N=555 )	Worse values at baseline (Elliott et al., 2018)
	SELs over time	RR (N=52 )	Worsening over 2 years (Preziosa et al., 2021)
		RR (N=1334 ), PP (N=555 )	Worsening over 96 and 120 weeks (Elliott et al., 2018)
		RR (N=83 )	Worsening over 3.2 years (Calvi, Tur, et al., 2022)
MP2RAGE (axonal damage)	SELs to non-SELs	RR (N=50 )	Worse values at baseline (Vavasour et al., 2025)
	SELs over time	RR (N=50 )	Worsening over 96 and 192 weeks (Vavasour et al., 2025)
	Non-SELs in SEL+ patients to non-SELs in SEL- patients	RR (N=50 )	Worse baseline values (Vavasour et al., 2025)
	NAWM in SEL+ patients to NAWM in SEL- patients	RR (N=50 )	More voxels had worse values and more deterioration over 96, 192 weeks (Vavasour et al., 2025)

The information in parentheses in the column “Metric used” indicates what tissue damage is the most associated with the metric in white matter, although the specificity is often low. The “Comparison” column indicates either what is evaluated and to what it is compared or what evolution has been investigated.

NAWM: normal appearing white matter, SEL: slowly expanding lesion, RD: radial diffusivity, AD: axial diffusivity, FA: fractional anisotropy, RR: relapsing-remitting, PP: primary progressive, SP: secondary progressive.

Overall, these studies demonstrated increased demyelination in SELs compared with non-SELs at baseline (Calvi et al., 2025; Elliott et al., 2020; Huerta et al., 2024; Klistorner, Barnett, Yiannikas, et al., 2022; Nakamura et al., 2024; Preziosa et al., 2021; Ravano et al., 2024; Vavasour et al., 2025) and more demyelination accumulation in SELs over time (Calvi, Tur, et al., 2022; Calvi et al., 2025; Elliott et al., 2020; Huerta et al., 2024; Klistorner, Barnett, Yiannikas, et al., 2022; Nakamura et al., 2024; Vavasour et al., 2025). By contrast, one study did not find any demyelination accumulation over 2 years (Preziosa et al., 2021). More demyelination was found in definite SELs than in possible SELs but possible SELs were associated with more variability in demyelination (Calvi, Tur, et al., 2022). SELs in pwSPMS had more demyelination than in pwRRMS at baseline, but there was no difference in demyelination evolution over time (Elliott et al., 2020). Most studies also found more axonal damage in SELs than in non-SELs at baseline (Calvi et al., 2025; Elliott et al., 2018; Preziosa et al., 2021; Vavasour et al., 2025) and more axonal damage accumulation in SELs over time (Calvi, Tur, et al., 2022; Calvi et al., 2025; Elliott et al., 2018; Klistorner, Barnett, Yiannikas, et al., 2022; Preziosa et al., 2021; Vavasour et al., 2025). However, one study (Huerta et al., 2024) found no difference in axonal damage at baseline and no accumulation over time in SELs. The black holes (BHs) are a potential marker of axonal loss (Sahraian et al., 2009). A first study found that 96% of SELs were BHs, versus 71% of non-SELs (Huerta et al., 2024), and another one that 61% of possible SELs, 52% of definite SELs, and 44% of non-SELs were persistent BHs (Calvi, Tur, et al., 2022). At the patient level, there was an association between SELs count and volume and baseline persistent BH count (Calvi, Tur, et al., 2022) and an association between higher SELs volume and more newly appearing persistent BHs over 96 weeks (Calvi, Carrasco, et al., 2022).

SELs periplaques had worse and more variable demyelination than stable lesion periplaques (Ravano et al., 2024). They also had more baseline axonal damage and accumulation (Calvi et al., 2025), but this result was not confirmed in another study (Klistorner, Barnett, Yiannikas, et al., 2022). In one study, axial diffusivity (AD) and radial diffusivity (RD) increased as much as each other in the cores, but RD increased substantially more than AD in the periplaques (Klistorner, Barnett, Yiannikas, et al., 2022). Moreover, there was no increase of diffusion in non-expanding lesions, and it was very isotropic in cores, but not in periplaques (Klistorner, Barnett, Yiannikas, et al., 2022).

Concerning the normal appearing white matter (NAWM) of SEL+ patients, more voxels showed abnormal demyelination and axonal damage at baseline and deterioration overtime than in SEL- patients in one study (Vavasour et al., 2025). Another study, however, found no correlation between expanding lesions and diffusivity in NAWM (Klistorner, Barnett, Yiannikas, et al., 2022), and one study found a moderate correlation between NAWM fractional anisotropy (FA) and baseline possible SELs and non-SELs FA, but not for definite SELs (Calvi et al., 2025). Non-SELs in SEL+ patients had more damage than non-SELs in SEL- patients (Vavasour et al., 2025).

#### 3.6.2 Microstructural damage as predictor of slow expansion

Two studies investigated whether damage measures could predict future lesion expansion (Klistorner, Barnett, Parratt, Yiannikas, & Klistorner, 2024b; Klistorner, Barnett, Yiannikas, et al., 2022). One found an association between pre-baseline and baseline RD (but not AD) in periplaque white matter and future expansion (Klistorner, Barnett, Yiannikas, et al., 2022). The other found a robust correlation between 2-year change in patient-wise mean diffusivity in expanding lesions and long-term (4 years) patient-wise volume expansion of SELs (Klistorner, Barnett, Parratt, Yiannikas, & Klistorner, 2024b). This correlation was only weak using T1w intensity and the correlation with central brain atrophy was higher.

One study (Ravano et al., 2024) explored the ability of lesion damage measurements at the last follow-up to infer lesion classes that reflect the evolution of lesion volume over time: stable, new, enlarging, or shrinking. The covariates were the standard deviation and mean of T1 relaxation time (MP2RAGE), T2 relaxation time (GRAPPATINI), and their ratio, within the lesion and in the perilesional rings. The overall accuracy was 73% and the most important covariates were the standard deviations of the measures.

**Statement:** Several studies show that at the group-scale SELs exhibit, at baseline, greater demyelination and more pronounced axonal loss, and a higher accumulation of microstructural damage over time than non-SELs. By contrast, findings are more heterogeneous regarding the severity of microstructural alterations in the periplaque and in the NAWM of patients with SELs. Similarly, the association between the initial severity of lesion damage and the subsequent evolution of a lesion into an SEL remains poorly investigated.

### 3.7 Associations between SELs and imaging biomarkers extracted from conventional MRI

#### 3.7.1 SELs and gadolinium enhancement

The proportion of voxels showing gadolinium enhancement was higher in non-SELs than in SELs (1.5% versus 0.3%) (Elliott et al., 2018), indicating a lack of strong blood–brain barrier disruption within SELs.

#### 3.7.2 SELs and brain atrophy

Brain atrophy and its derivatives are imaging biomarkers associated with neurodegeneration. One study investigating baseline brain parenchymal fraction found an association with higher SELs volume (Calvi et al., 2023).

In studies investigating atrophy measured using the same baseline and follow-up scans as for SELs, whole-brain atrophy was correlated with definite SELs volume (Calvi, Carrasco, et al., 2022), FA changes in the perilesional areas, and cores of SELs (Calvi et al., 2025), but not possible SELs nor non-SELs (Calvi, Carrasco, et al., 2022). SELs count was associated with cortical and subcortical gray matter atrophy (Yokote et al., 2024). The quartile of patients with the highest SELs volume had a higher atrophy rate, especially central brain atrophy (ventricular enlargement), even adjusting for the volume of new lesions (Klistorner et al., 2025). SELs volume was highly correlated with central brain atrophy, the correlation was even stronger using SELs PVSI (lesion volume multiplied by a measure of tissue integrity change within the lesion), and these correlations were higher than with whole-brain atrophy (Klistorner, Barnett, & Klistorner, 2022).

Conversely, 80% of lesion expansion over 4 years can be explained by 2 years central-brain atrophy (Klistorner, Barnett, Parratt, Yiannikas, & Klistorner, 2024b). Moreover, central-brain atrophy could predict whether a patient will have long-term (4-year) SELs expansion with 94% sensitivity and 85% specificity (Klistorner, Barnett, Parratt, Yiannikas, & Klistorner, 2024b).

#### 3.7.3 SELs and choroid plexus volume

The choroid plexus (CP) plays an important role in the brain immune response and is essential for the production of CSF (Okar et al., 2024). Baseline CP volume was strongly correlated with SELs rate of expansion and SELs damage, as measured by mean diffusivity (MD) (Klistorner, Barnett, et al., 2022). Baseline CP volume could predict whether a patient will have long-term expansion, with 85% sensitivity and 76% specificity (Klistorner, Barnett, et al., 2022). CP enlargement was associated with SELs volume change and SELs damage and PVSI, even adjusting for new lesions volume (Klistorner, Barnett, Wang, et al., 2024).

**Statement:** Numerous studies have highlighted a link between the number and volume of SELs and the development of brain atrophy, both global and involving only cortical and deep gray matter, and ventricular enlargement. SELs showed association with choroid plexus volume, although this association was investigated in only a few studies. Gadolinium enhancement was rarely observed in SELs, but was investigated in only one study.

### 3.8 Associations between SELs and biological markers

In this section, we explore the relationships between SELs and biological biomarkers extracted from the cerebro-spinal fluid (CSF), the plasma, and the serum. Detailed results of the studies are provided in Table 2.

**Table 2.** Associations between SELs and biological markers of the CSF, the plasma, and the serum.

Biological marker	Specificity	Disease phenotype	Observation
Serum+plasma NfL	Neuroaxonal injuries	RR (N=1421 ), PP (N=596 )	High baseline levels were associated with greater SELs count, baseline volume, volume increase, and T1w intensity decrease (Bar-Or et al., 2023)
Serum GFAP	Astrocyte activity	SP (N=264 )	No significant associations between baseline serum GFAP and SELs volume and volume changes (Jiang et al., 2023)
CSF NfL	Neuroaxonal injuries	RR (N=100 ), PP (N=31 )	Baseline levels were associated with SELs count (Cross et al., 2024)
CSF NfH	Neuroaxonal injuries	RR (N=100 ), PP (N=31 )	Baseline levels were associated with SELs count and lower T1w intensity (Cross et al., 2024)
CSF CXCL12	Chemokine	RR (N=100 ), PP (N=31 )	Baseline levels were associated with lower SELs T1w intensity (Cross et al., 2024)
CSF YKL-40	Astrocyte activity	RR (N=100 ), PP (N=31 )	Baseline levels were associated with SELs count (Cross et al., 2024)
CSF GFAP	Astrocyte activity	RR (N=100 ), PP (N=31 )	Baseline levels were associated with SELs count, baseline volume, and lower T1w intensity, even when adjusting for T2 lesion volume (Cross et al., 2024)

SELs: slowly expanding lesions, CSF: cerebro-spinal fluid, NfL: neurofilament light chain, NfH: neurofilament heavy chain, CXCL12: C-X-C motif chemokine 12, GFAP: glial fibrillary acidic protein, YKL-40: chitinase-3-like protein 1, RR: relapsing-remitting, PP: primary progressive, SP: secondary progressive.

With regard to serum and plasma markers, SELs metrics were associated with baseline NfL levels (Bar-Or et al., 2023), but not significantly associated with GFAP (Jiang et al., 2023). For CSF markers, one study considered the correlation between SELs and various baseline CSF biological measures (Cross et al., 2024). SELs metrics were associated with NfL, NfH, YKL-40, CXCL12, and GFAP (Cross et al., 2024). No significant correlation was found between these SELs metrics and a marker of astrocyte activity/inflammation (LCN2), B/T cell markers (CD19 + B cells, CD3 + T cells, sTACI, sCD27, sBCMA), microglia activity (sTREM 2), cytokines (IL-6), or chemokines (CXCL13, CXCL10, CCL19), except one chemokine (CXCL12) (Cross et al., 2024). The two results for GFAP are not contradictory, as the correlation between serum GFAP and CSF GFAP is only moderate (Cross et al., 2024).

**Statement:** Associations between SELs metrics and several CSF and serum markers of neuroaxonal injuries and astrocyte activity are reported in only a few studies. Independent studies are required for further validation. The relationship between SELs and other biological measurements needs to be investigated.

### 3.9 Associations with clinical metrics

Associations were studied between SELs and various clinical metrics: the Expanded Disability Status Scale (EDSS), the nine-hole peg test (9HPT), the timed 25-foot walk test (T25FWT), the paced auditory serial addition test (PASAT), and the symbol digit modalities test (SDMT).

SELs metrics were associated with baseline disability measures: EDSS (Calvi et al., 2025; Klistorner et al., 2025), SDMT (Nakamura et al., 2024), 9HPT (Nakamura et al., 2024), and T25FWT (Nakamura et al., 2024). However, one study found no correlation between SELs metrics and baseline EDSS (Nakamura et al., 2024).

SELs metrics were also associated with the longitudinal evolution of clinical measures of disability, as detailed in Table 3. SELs were associated with progression of EDSS (Beynon et al., 2022; Calvi, Carrasco, et al., 2022; Calvi, Tur, et al., 2022; Calvi et al., 2023, 2025; Klistorner et al., 2025; Preziosa et al., 2022; Yokote et al., 2024), T25FWT (Beynon et al., 2022; Calvi, Carrasco, et al., 2022; Nakamura et al., 2024), 9HPT (Beynon et al., 2022; Calvi, Carrasco, et al., 2022; Nakamura et al., 2024), PASAT (Calvi, Carrasco, et al., 2022), SDMT (Calvi, Carrasco, et al., 2022), combined disability progression (EDSS+T25FWT+9HPT) (Beynon et al., 2022; Elliott et al., 2019), (PASAT+T25FWT+9HPT) (Calvi, Carrasco, et al., 2022; Calvi et al., 2025), and long-term conversion from RRMS to SPMS (Preziosa et al., 2022). However, one study found no correlation between SELs volume and EDSS progression in pwPMS, although SELs volume was correlated with EDSS progression quartile groups (Nakamura et al., 2024). Another study found that 9HPT change was not associated with SELs volume in SPMS (Calvi, Carrasco, et al., 2022). These associations have repeatedly been shown to be significant even adjusting for baseline lesion burden (Calvi, Tur, et al., 2022; Calvi et al., 2023, 2025; Nakamura et al., 2024; Preziosa et al., 2022) and brain atrophy (Calvi, Carrasco, et al., 2022; Calvi, Tur, et al., 2022; Calvi et al., 2025), and even if there was no recent or ongoing acute inflammatory activity (Beynon et al., 2022; Yokote et al., 2024).

**Table 3.** Effect of SELs metrics on clinical measures of disability.

Clinical measure of disability	Disease phenotype	Observation
EDSS	RR (N=72 )	The quartile of patients who had the most SELs expansion over 5 years had a higher baseline EDSS and 5-year EDSS worsening (Klistorner et al., 2025)
	RR (N=135 )	SELs volume was associated with a 3.2-year progression (Calvi, Tur, et al., 2022)
	RR (N=52 )	Baseline SELs MTR and SELs relative volume were predictors of 9 years worsening, but not T1w intensity (Preziosa et al., 2022)
	RR (N=61 )	SELs volume was associated with progression over a median of 3.2 years, SEL+/PRL+ patients progressed the most (Calvi et al., 2023)
	SP (N=345 )	SELs volume was associated with progression over 96 weeks (Calvi, Carrasco, et al., 2022)
	SP (N=600 )	Patients who progressed over 108 weeks had a more severe T1w change in SELs (Beynon et al., 2022)
	PP (N=107 ), SP (N=88 )	SELs volume was not correlated with baseline EDSS, nor with disability progression over 96 weeks, but SELs volume was correlated with EDSS quartile groups (Nakamura et al., 2024)
	RR+PP+SP (N=99 )	SELs count was associated with losing NEDA-3 status over 24 months (Yokote et al., 2024)
	RR+PP+SP (N=130 )	FA changes within SELs cores and perilesional areas predicted a higher risk and faster progression, independently of relapse activity (Calvi et al., 2025)
T25FWT	SP (N=345 )	SELs volume explained a proportion of worsening over 96 weeks (Calvi, Carrasco, et al., 2022)
	SP (N=600 )	Patients who progressed over 108 weeks had a more severe T1w change in SELs (Beynon et al., 2022)
	PP (N=107 ), SP (N=88 )	SELs volume was correlated with baseline T25FWT (Nakamura et al., 2024)
	PP (N=107 ), SP (N=88 )	Definitely expanding patients had a worse change after 96 weeks (Nakamura et al., 2024)
9HPT	SP (N=345 )	SELs volume did not explain any worsening over 96 weeks (Calvi, Carrasco, et al., 2022)
	SP (N=600 )	Patients who progressed over 108 weeks had a more severe T1w change in SELs (Beynon et al., 2022)
	PP (N=107 ), SP (N=88 )	SELs volume was correlated with baseline 9HPT (Nakamura et al., 2024)
PASAT	SP (N=345 )	SELs volume was associated with progression over 96 weeks (Calvi, Carrasco, et al., 2022)
SDMT	SP (N=345 )	SELs volume was associated with a worsening over 96 weeks (Calvi, Carrasco, et al., 2022)
	PP (N=107 ), SP (N=88 )	SELs volume was correlated with baseline SDMT (Nakamura et al., 2024)
CDP	PP (N=732 )	T1w intensities in 120-week SELs predicted progression between week 120 and week 240 (EDSS, T25FWT, and 9HPT) and were more predictive than baseline T1 volume burden (Elliott et al., 2019)
	SP (N=345 )	SELs volume predicted progression (9HPT, T25FW, and PASAT) over 96 weeks (Calvi, Carrasco, et al., 2022)
	SP (N=600 )	Patients who progressed (EDSS, T25FWT, and 9HPT) over 108 weeks had a more severe T1w change in SELs (Beynon et al., 2022)
SP conversion	RR (N=52 )	Baseline SELs MTR and T1w intensity decline predicted 9-year conversion to SPMS (Preziosa et al., 2022).

EDSS: expanded disability status scale, 9HPT: nine-hole peg test, T25FWT: timed 25-foot walk test, PASAT: paced auditory serial addition task, SDMT: symbol digit modalities test, CDP: combined disability progression, NEDA-3: no evidence of disease activity for 3 months, RR: relapsing-remitting, PP: primary progressive, SP: secondary progressive, SEL: slowly expanding lesion, PRL: paramagnetic rim lesion, FA: fractional anisotropy.

**Statement:** Numerous studies support an association between SELs and baseline disability scores, and between SELs and longitudinal increases in disability scores. Various studies showed these longitudinal associations to be independent from brain lesion load and atrophy. These findings highlight the clinical relevance of this biomarker.

### 3.10 Effect of the treatments

The effects of various DMTs on SELs have been examined in only a small number of studies. Their results are compiled in Table 4. Ibudilast (Nakamura et al., 2024), natalizumab (Beynon et al., 2022), and ocrelizumab (Elliott et al., 2019) were reported to moderately reduce SELs metrics compared with placebo. Evobrutinib also moderately reduced SELs metrics, in a dose-dependent way (Arnold et al., 2024). In a comparison of two high-efficacy treatments, natalizumab had a higher effect on SELs than fingolimod (Preziosa et al., 2021). For patients under high- (natalizumab or fingolimod) and moderate-efficacy treatments (injectables), having at least two SELs was a risk factor of disability progression without relapses or new lesions, independent of the treatment group (Yokote et al., 2024), indicating a low effect on SELs. In a comparison of a high- and a moderate-efficacy treatment, ocrelizumab reduced damage in SELs, but not the proportion of patients with at least one SEL, compared with interferon β-1a (Vavasour et al., 2025).

**Table 4.** Effects of treatments on SELs.

Disease-modifying therapy	Reference therapy	Treatment repartition	Disease phenotype	Observation
Evobrutinib (25 mg/day, 75 mg/day, 2 ×75mg/day)	Placebo for 24w then evobrutinib 25 mg/day	(1:1:1:1)	RR (N=228 ), SP (N=33 )	48-week SELs absolute and relative volume were reduced in a dose-dependent way, but there was no effect on SELs count (Arnold et al., 2024)
Natalizumab	Placebo	(1:1)	SP (N=600 )	SELs baseline volume, relative and absolute T1w volume change over 100 weeks, and absolute and relative counts were reduced (Beynon et al., 2022)
Ibudilast	Placebo	(1:1)	PP (N=107 ), SP (N=88 )	SELs volume and MTR change decreased over 96 weeks; the effect on MTR was small (0.22/year) compared with the random effect’s standard deviation of 4.4 (Nakamura et al., 2024)
Natalizumab	Fingolimod	(1:1)	RR (N=52 )	SELs baseline absolute and relative volume and count were moderately reduced (Preziosa et al., 2021)
Ocrelizumab	Placebo	(2:1)	PP (N=732 )	The relative change in T1w hypointense lesion volume of SELs over 120 weeks was reduced (Elliott et al., 2019)
Ocrelizumab	Interferon β-1a	(1:1)	RR (N=50 )	96-week SELs MTR change was reduced, as many patients had at least one SEL in each treatment group (Vavasour et al., 2025)
High efficacy (natalizumab or fingolimod)	Moderate efficacy (injectables)	(1:1)	RR (N=99 )	Having two 2-year SELs was a risk factor of disability progression without relapses or new lesions independent of the treatment group (Yokote et al., 2024)

SELs: slowly expanding lesions, MTR: magnetization transfer imaging, RR: relapsing-remitting, PP: primary progressive, SP: secondary progressive.

**Statement:** The effects of various DMTs, compared with placebo or with other DMTs, on SELs were evaluated using imaging data from therapeutic trials. Independent studies using data acquired in clinical practice are required for further validation. Evidence of a treatment effect on SELs, albeit often modest, was observed. These findings support the notion that SELs may constitute a promising biomarker for assessing the impact of novel therapies on the chronic inflammatory component of MS. Moreover, as SELs can be extracted from MRI scans acquired during routine care, this biomarker can also be used in real-world treatment-efficacy studies, with longer-term follow-up.

## 4 Discussion

### 4.1 Scoping review process

The inclusion process allowed us to identify a relatively large number of articles reporting the detection of SELs in pwMS. However, this process is limited by the heterogeneity of terminology used to describe SELs, which may not have been fully captured by our search strategy. In addition, the search may have missed studies in which SELs were analyzed alongside other imaging markers, without being the primary focus. To mitigate these limitations, we systematically screened the reference lists of the included articles; however, no additional eligible studies were identified through this process.

### 4.2 Limitations and future perspectives

SEL detection currently faces several limitations. First, as detailed in the methodological section of this review, the detection methods are not fully standardized, and critical parameters may vary between studies, resulting in limited inter-study comparability. Studies evaluating the impact of methodological choices (e.g., segmentation versus deformation methods) and threshold selection are required to improve both SEL detection and cross-study consistency. In addition, making SEL identification pipelines openly available online would facilitate a wider adoption of this method and enable validation on diverse datasets acquired in routine clinical practice. To date, most published studies have relied on imaging data obtained within therapeutic trials, while large cohort studies using routine care imaging data are lacking. Such cohort would enable the investigation of the impact of different therapeutic strategies on SELs in real-world settings, and the study of various comorbidities potentially associated with SELs, such as cardiovascular risk factors.

Furthermore, the sensitivity and specificity of SELs as a biomarker of chronic active lesions remain to be established. The modest co-occurrence observed between SELs and PRLs suggests that these measures may capture different aspects of chronic active lesions and distinct stages of their evolution. Studies combining SELs with TSPO PET imaging, and postmortem histopathological analyses, could provide valuable insights into their biological underpinnings and help refine their specificity and sensitivity.

Another important limitation is that current methods require longitudinal data over at least two time points, typically spanning 1 year in clinical practice. Consequently, this imaging biomarker cannot be assessed at diagnosis, unlike PRLs, which can be detected from a single scan. While PRLs and SELs provide complementary information, the added value of their combined evaluation in longitudinal clinical follow-up remains to be determined. Moreover, current approaches do not account for scanner changes, as brain volume measures are not preserved across scanners. Addressing the impact of scanner variability and developing methods to correct for it will be crucial for successful clinical translation.

Finally, SEL occurrence has not yet been systematically investigated in the spinal cord and optic nerves. Existing methods must be adapted to determine whether SELs can be characterized in these regions and how they relate to disability progression.

## 5 Conclusion

The studies in this review consistently show that SELs are a common feature of MS, present in most MS patients, for every disease phenotype, and representing a large proportion of the T2 lesion load. Additionally, they show that SELs are a relevant biomarker in MS that displays many relationships with other imaging and biological biomarkers. While the effect of treatments on SELs was weak, the various comparisons indicate that SELs could be a relevant biomarker for evaluating the effects of treatments on chronic inflammation. Finally, numerous studies consistently demonstrate that SELs are associated with baseline disability scores and their longitudinal increase, often independently from other common imaging biomarkers known to be associated with disability progression such as brain atrophy and lesion load. This strongly indicates the potential clinical value of SELs as a biomarker of disability progression. As well as these promising results, our review outlines several limitations including the lack of method standardization, limiting inter-study comparability, and the lack of knowledge about what SELs are to CALs, notably about their specificity and sensitivity.

## Data Availability

The data concerning the query results for the paper selection are available from the corresponding author, M.G., upon request. No other new data were created or analyzed in this study.

## A Appendix

### A.1 Sels Score

Lesions classified as expanding lesions may not specifically be slowly expanding, as new lesions can appear next to a pre-existing lesion between baseline and follow-up, and as baseline lesions that are still active also enlarge, but rapidly and for a shorter duration. Elliott et al. (2018) sought to tackle this issue for the deformation-based analysis, using scans acquired at intermediate time points. The first scenario should reduce the isotropy and concentricity of the deformation and both scenarios should induce a less constant expansion. To ensure that an SEL candidate has a concentric and constant expansion, they assigned two scores:

- **Concentricity score:** The lesion is first divided into concentric bands of equal distance from the lesion edge, and the mean Jacobian determinant in each band is calculated; the concentricity score is defined as the slope of least-squares linear fit of the mean Jacobian determinant in each band according to the distance of the bands to the edge (Calvi, Carrasco, et al., 2022).
- **Constancy score:** An SEL expansion over time can be determined using the Jacobian determinant between the baseline and each follow-up time point, determined in Step 3 (Section 3.2). The constancy score is the mean squared error of the linear regression without the intercept term of the expansion over time (Elliott et al., 2018). For an SEL, considering the n follow-up time points $\text{t}=\left(t_{1},...,t_{n}\right)$ and the n associated lesion expansions since the baseline $\text{x}=\left(x_{1},...,x_{n}\right)$. The rate of expansion is given by β∈ℝ minimizing the sum of the squared residuals $\varepsilon =\left(\varepsilon_{1},...,\varepsilon_{n}\right)$, such that

x=βt+ε.

The best fit normalized mean squared error, or constancy score, is given by

$$ê=\frac{1}{n}\sum_{i=1}^{n}{\left(\frac{x_{i}-\beta t_{i}}{\beta t_{i}}\right)}^{2}.$$

Then, the mean and standard deviations of both scores are derived from a cohort, often the whole studied patient population, to z-normalize them before adding them together to form the SEL heuristic score (Elliott et al., 2018). Noting $\hat{c}$ the concentricity score, $\mu_{c},\mu_{e},\sigma_{c},\sigma_{e},$ respectively, the mean and standard deviations of the concentricity and constancy scores, the heuristic score is given by

$$\frac{\hat{c}-\mu_{c}}{\sigma_{c}}-\frac{ê-\mu_{e}}{\sigma_{e}}.$$

“Definite SELs” are defined as SELs with a positive heuristic score and the others are “possible SELs”. A representation of the scores is available in Elliott Colm’s paper (Elliott et al., 2018), Figure 2.

It must be noted that the estimator of the constancy of the expansion over time ê is numerically unstable as it tends to infinity as β tends to zero, which could for instance happen on occasions where the lesion first shrinks through remyelination and then artificially grows because of a new lesion appearing next to it. Very large score value would largely influence the estimation of the mean and standard deviation of the constancy score.

### A.2 Results of Sels Score

A comparison between all SELs and definite SELs (Elliott et al., 2019) showed that definite SELs had a slightly higher mean T1w intensity decrease than all SEL candidates. Furthermore, changing the heuristic threshold had no significant impact on the differentiation between patients under placebo or under high-efficacy treatments (ocrelizumab), shown by the fact that the treatment had a significant impact over T1w hypointense volume accumulation above all tested heuristic thresholds. Moreover, the prevalence of definite SELs and all SELs in RRMS versus PPMS was similar, with a median all SELs count ratio of 5.1% for PPMS and 7.1% for RRMS versus a median definite SELs count of 2.5% and 3.4%, respectively. In another study, the mean volume change per year was 3% for definite SELs and 1.5% for possible SELs (it was also 1.5% for non-SELs) (Calvi, Carrasco, et al., 2022); definite SELs volume was associated with brain atrophy, but possible SELs volume was not and possible SELs correlated better with the progression than definite SELs (Calvi, Carrasco, et al., 2022). No difference in the distribution of brain lesion location was found between definite and possible SELs (Calvi, Carrasco, et al., 2022).
